# Wearable based monitoring and self-supervised contrastive learning detect clinical complications during treatment of Hematologic malignancies

**DOI:** 10.1038/s41746-023-00847-2

**Published:** 2023-06-02

**Authors:** Malte Jacobsen, Rahil Gholamipoor, Till A. Dembek, Pauline Rottmann, Marlo Verket, Julia Brandts, Paul Jäger, Ben-Niklas Baermann, Mustafa Kondakci, Lutz Heinemann, Anna L. Gerke, Nikolaus Marx, Dirk Müller-Wieland, Kathrin Möllenhoff, Melchior Seyfarth, Markus Kollmann, Guido Kobbe

**Affiliations:** 1 Faculty of Health, University Witten/Herdecke, 58448 Witten, Germany; 2 Department of Internal Medicine I, University Hospital Aachen, RWTH Aachen University, 52074 Aachen, Germany; 3 Department of Computer Science, Heinrich Heine University Düsseldorf, 40225 Düsseldorf, Germany; 4 Department of Neurology, Faculty of Medicine, University of Cologne, 50937 Cologne, Germany; 5 Department of Hematology, Oncology, and Clinical Immunology, University Hospital Düsseldorf, Medical Faculty, Heinrich Heine University Düsseldorf, 40225 Düsseldorf, Germany; 6 Department of Oncology and Hematology, St. Lukas Hospital Solingen, 42697 Solingen, Germany; 7 Science-Consulting in Diabetes, 41564 Kaarst, Germany; 8 Mathematical Institute, Heinrich Heine University Düsseldorf, 40225 Düsseldorf, Germany; 9 Department of Cardiology, Helios University Hospital Wuppertal, 42117 Wuppertal, Germany; 10 Department of Biology, Heinrich Heine University Düsseldorf, Düsseldorf, 40225 Germany

**Keywords:** Medical research, Diagnosis

## Abstract

Serious clinical complications (SCC; CTCAE grade ≥ 3) occur frequently in patients treated for hematological malignancies. Early diagnosis and treatment of SCC are essential to improve outcomes. Here we report a deep learning model-derived SCC-Score to detect and predict SCC from time-series data recorded continuously by a medical wearable. In this single-arm, single-center, observational cohort study, vital signs and physical activity were recorded with a wearable for 31,234 h in 79 patients (54 Inpatient Cohort (IC)/25 Outpatient Cohort (OC)). Hours with normal physical functioning without evidence of SCC (regular hours) were presented to a deep neural network that was trained by a self-supervised contrastive learning objective to extract features from the time series that are typical in regular periods. The model was used to calculate a SCC-Score that measures the dissimilarity to regular features. Detection and prediction performance of the SCC-Score was compared to clinical documentation of SCC (AUROC ± SD). In total 124 clinically documented SCC occurred in the IC, 16 in the OC. Detection of SCC was achieved in the IC with a sensitivity of 79.7% and specificity of 87.9%, with AUROC of 0.91 ± 0.01 (OC sensitivity 77.4%, specificity 81.8%, AUROC 0.87 ± 0.02). Prediction of infectious SCC was possible up to 2 days before clinical diagnosis (AUROC 0.90 at −24 h and 0.88 at −48 h). We provide proof of principle for the detection and prediction of SCC in patients treated for hematological malignancies using wearable data and a deep learning model. As a consequence, remote patient monitoring may enable pre-emptive complication management.

## Introduction

Treatment of patients with hematological malignancies is associated with a high incidence of clinical complications, such as infections, cardiac events, and immunologic dysregulations^1,2^. These potentially life-threatening complications require early recognition and therapeutic intervention, as it is known that delayed intervention is associated with increased morbidity and mortality^3,4^. Recent diversification of oncological treatment options, including e.g. CAR-T cell therapy, increase therapeutic options but add to the spectrum of complications, such as ‘cytokine release syndrome’. Today’s management of complications depends on the setting of oncological treatment: Under hospital conditions - referred to as inpatient setting-the management of complications relies on intermittent recordings of vital signs, daily clinical examinations, and laboratory tests by health care professionals (HCP). However, an increasing number of oncological treatments are applied in the outpatient setting^5^, where complication detection relies primarily on patient self-assessment^6^. Early detection of (subtle) symptoms indicating complications is challenging and is often delayed. To avoid ‘late show ups’, outpatients are routinely admitted to their treatment center without evidence of complications, which burdens patients and HCP^7^. Therefore, there is a need for innovative concepts for early and reliable detection of treatment-associated complications^8^.

Remote patient monitoring (RPM) with medical wearables represents a novel option for non-invasive and continuous real-time monitoring of vital signs and physical activity^9–11^. Medical wearables provide longitudinal and high-resolution health data that expand monitoring options and allow real-time complications detection by classification models^12,13^. For automated classification, the recorded datasets should be pooled across all patients to increase the statistical power of machine learning models, which frequently show superior performance for large data sets compared to classifiers that use hand-engineered features. However, with a single classification model at hand, the challenge remains of how to adjust the classification threshold for each patient. To address this challenge, self-supervised feature learning from all patient data was combined with a similarity score to assess proximity to patient-specific features. Similar concepts have been successfully employed for anomaly detection in the visual and audio domains^14,15^.

Here we report that a wearable-based RPM approach in combination with a self-supervised contrastive deep learning model can sufficiently detect and predict serious clinical complications (SCC) for in- and outpatients during their oncological treatment for hematological malignancies.

## Results

### Performance of SCC-score

For the patient-non-specific and patient-specific approach SCC-Scores were significantly higher in non-regular hours and days, indicating a higher risk for SCC prevalence compared to sets of regular hours (Table 2). This observation was stable with ten-fold cross-validation (Supplementary Fig. 3).

**Fig. 1:**
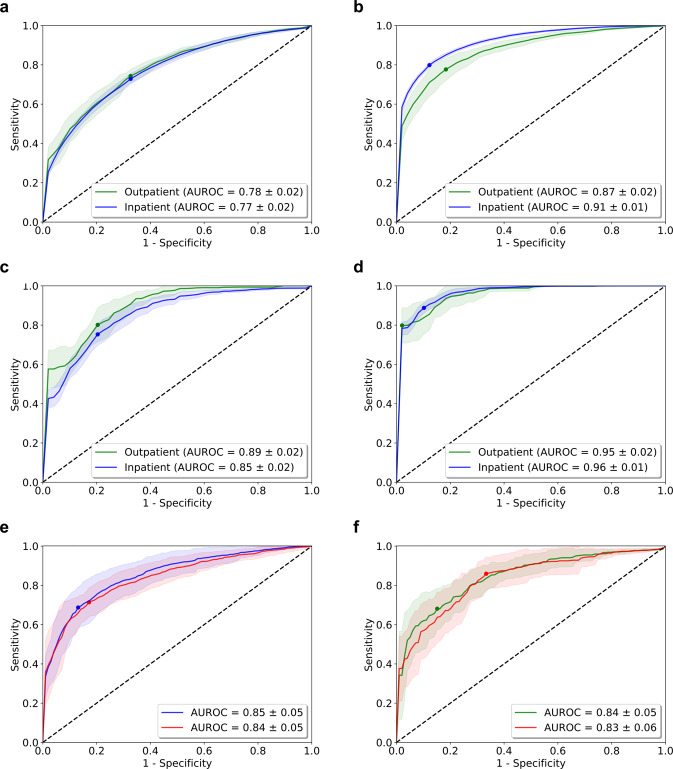
Performance of the SCC-Score detecting SCC. The area under the Receiver Operating Characteristic curves (AUROC) for the IC (blue line) and OC (green line) using both hourly and per-day SCC-Scores, in the patient-non-specific approach (**a**, **c**) and with the patient-specific approach (**b**, **d**). AUROC curves using the patient-non-specific approach are given for patient #1001 (**e**) from the inpatient cohort (blue) and #1067 (**f**) from the outpatient cohort (green) showing individual performance of the SCC-Score. Red lines (in e and f) show the performance when the specific data of the patients was excluded during training.The dots on the lines mark the cut-point that optimizes the detection of sensitivity and 1-specificity (Youden index). Standard errors of the AUROC curves are computed from ten-fold cross-validation are given in shaded areas.

**Fig. 2:**
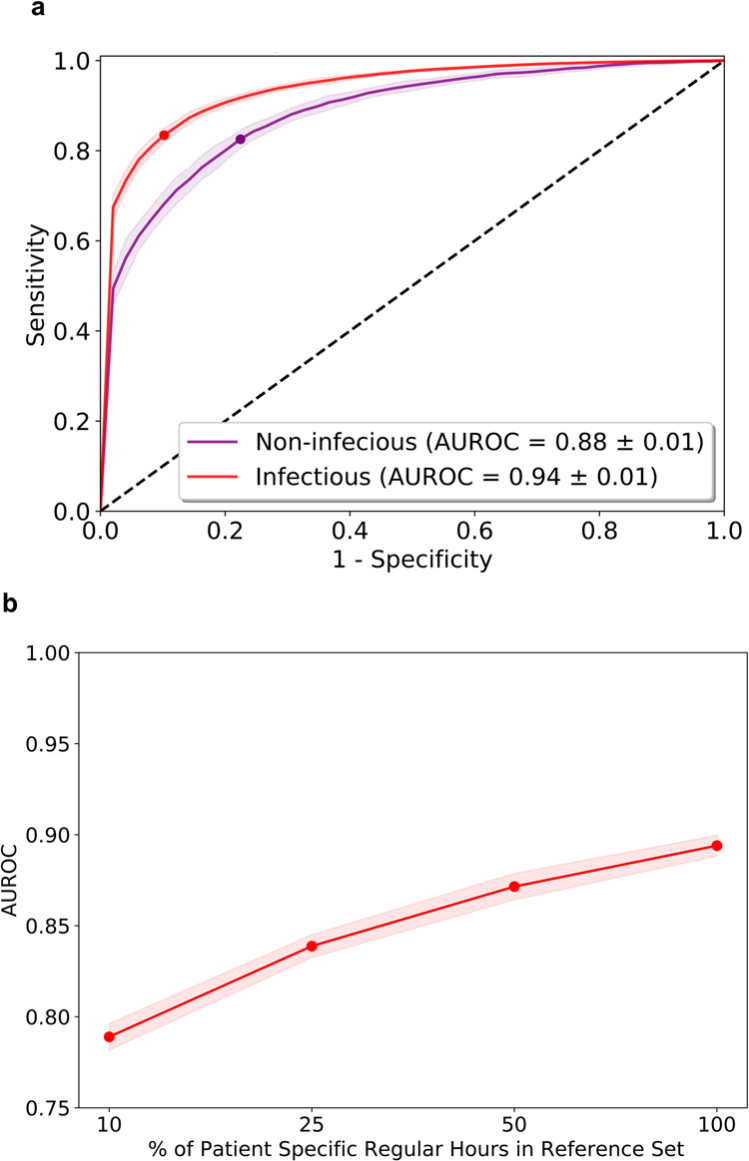
Subanalysis of SCC-Score performance. **a** Area under the Receiver Operating Characteristic curves (AUROC) using hourly SCC-Score with the patient-specific approach for infectious (red line) vs. non-infectious (purple line) for the total. Standard errors of the AUROC curves are computed from ten-fold cross-validation and show as shaded areas. **b** Performance of the SCC-Score with increasing percentage of specific regular hours in the reference set (red line).

The performance in the patient non-specific approach showed an average AUROC for IC of 0.77 and OC 0.78 (Fig. 1a). A significant increase in performance for the patient-specific approach was observed (IC 0.87 and OC 0.91, Fig. 1b). A per-day SCC-Score further increased the AUROC by ~10% (IC 0.85 and OC 0.89, Fig. 1c, d). The per-day SCC-Score for the patient-specific approach resulted in the best estimate for SCC (Fig. 1d). For a randomly chosen patient from the IC and OC, AUROC values of 0.85 and 0.84 were observed, respectively (Fig. 1e, f). Excluding patient specific data of these patients during training revealed an equivalent performance. The per-day SCC-Score showed similar good performance if the annotation of SCC included the buffers of 48 h before and the 24 h after the documented SCC day (data not shown). Assessment of hourly SCC-Scores for the infectious vs. non-infectious SCC showed an 8.4% increase in AUROC pro-infectious (Fig. 2a). Performance of the SCC-Score increased with higher percentages of patient specific regular hours in the reference set (Fig. 2b).

### Prediction capabilities of SCC-Score

**Fig. 3:**
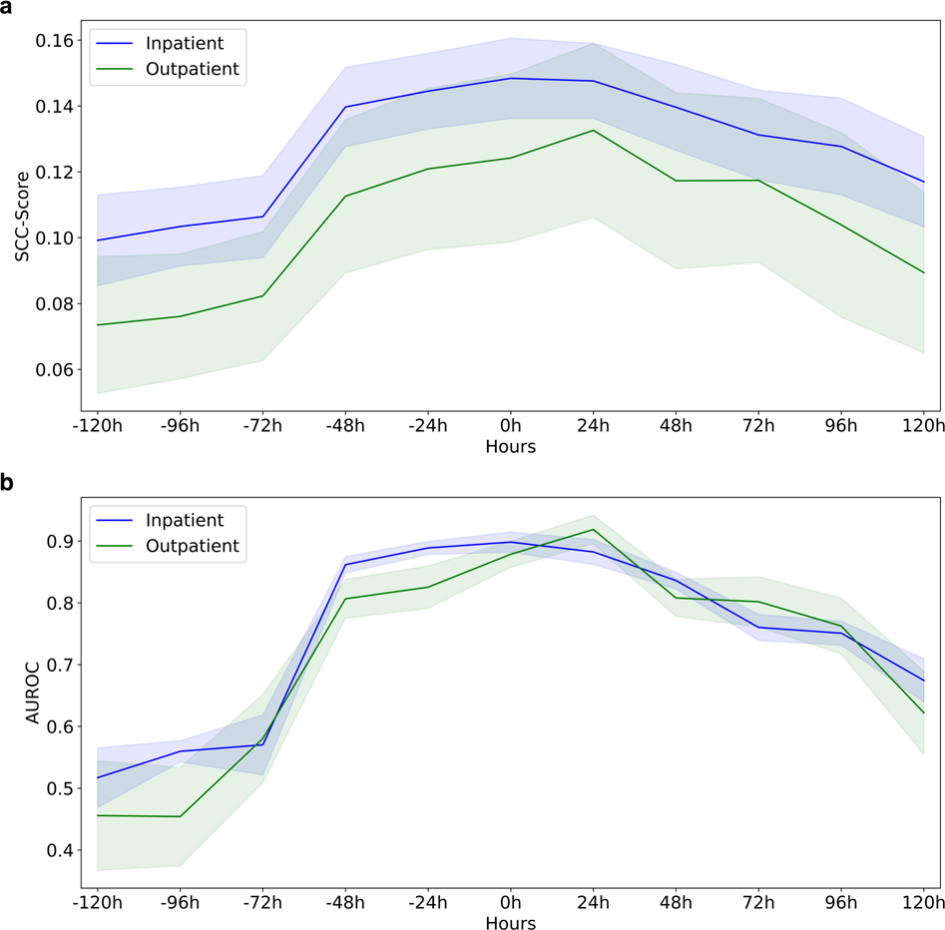
Prediction performance of the SCC-Score. Time dependence of the SCC-Score for infectious SCC. **a** Score values before and post-diagnosis at time point *t* = 0 h for the patients in the Inpatient Cohort, and Outpatient Cohort. Shaded areas indicate the standard deviation of SCC-Score values. **b** Prediction performance (AUROC) of hours containing infectious SCC based on SCC-Score. Standard errors are computed from ten-fold cross-validation and shown as shaded areas.

To evaluate the SCC-Score’s prediction capabilities over time, the time point of clinical diagnosis of infectious SCC was set to time *t* = 0 h. The SCC-Scores computed by the model for the hours before and after each documented SCC showed a transient increase followed by a transient decrease, attending high SCC-scores ~48 h before and 24 h after diagnosis (Fig. 3a and Supplementary Table 4). To visualize that both IC and OC follow the same transient behavior despite their difference in average SCC-Score values, the AUROC values were computed over time (Fig. 3b).

### Relationships of the SCC-Score

IC had a lower SCC-Score variance in regular hours than OC (−17.8%). The mean SCC-Score levels differed between the IC and OC, with IC scores 8.4% higher than OC scores. This difference in SCC-Score levels was more pronounced for infectious SCC, with IC scores 15.9% higher than OC scores. At a sensitivity of ~95.0% for the patient non-specific approach the hourly SCC-Score showed a low specificity (IC 22.2% and OC 22.2%). In contrast, only a moderate decline in specificity was observed (IC 56.6% and OC 41.4%) for the patient-specific approach.

The averaged z-score of ten-fold cross-validation for days with different types of SCC differ (Table 1; last column), with z-scores ranging from 1.00 for syncope (*n* = 2) to 3.28 for paroxysmal atrial tachycardia (*n* = 3). For the most common SCC (‘Infections and infestations—other’), the averaged z-score was 2.21 (*n* = 66).

**Table 1:** Specifications of Serious Clinical Complications (SCC).

No.	Adverse event	Criteria for Grade 3 in common terminology criteria for adverse events (CTCAE)	SCC [*n*]	z-score
1	Infections and infestations —other^a^	Severe or medically significant but not immediately life-threatening; hospitalization or prolongation of existing hospitalization indicated; disabling; limiting self-care ADL	66	2.21
2	Lung infection^a^	IV started	11	2.67
3	Hypertension	Stage 2 hypertension […]; medical intervention indicated […]	11	2.18
4	Mucositis oral^a^	Severe pain; interfering with oral intake	11	1.94
5	Nausea	Inadequate oral caloric or fluid intake, TPN	9	1.48
6	Pulmonary edema	diuretics indicated	3	2.28
7	Sinus tachycardia	Urgent medical intervention indicated	3	2.99
8	Allergic reaction	Prolonged […] and/or brief interruption of the infusion	3	1.52
9	Pain	Severe pain; limiting self-care ADL	3	2.64
10	Paroxysmal atrial tachycardia	IV medication indicated	3	3.28
11	Hypotension	Medical intervention or hospitalization indicated	2	2.42
12	Dyspnea	Shortness of breath at rest; limiting self-care ADL	2	1.79
13	Diarrhea	Increase of ≥7 stools per day over baseline	2	2.33
14	Syncope	Fainting; orthostatic collapse	2	1.00
15	Periorbital edema	Diuretics indicated	1	1.15
16	Oral pain	Severe pain; limiting self-care ADL	1	1.18
17	Colitis^a^	Severe abdominal pain […]; medical intervention indicated; peritoneal signs	1	1.02
18	Hypokalemia	<3.0–2.5 mmol/L; hospitalization indicated	1	1.53
19	Immune system disorders—other	Severe or medically significant but not immediately life-threatening; hospitalization […]	1	1.14
20	Cholecystitis^a^	Severe symptoms; radiologic, endoscopic, or elective operative intervention indicated	1	1.75
21	Catheter-related infection^a^	IV antibiotic, antifungal, antiviral, radiologic, or operative intervention indicated	1	2.77
22	Hepatobiliary disorders—other	Severe or medically significant but not immediately life-threatening; hospitalization […]	1	1.60
23	GGT increased	>5.0–20.0 x ULN	1	2.30

SCC were specified based on adverse events classification (Common Terminology Criteria for Adverse Events v4.0 (2009)) sorted in order of cumulative frequency of occurrence for Inpatient cohort (IC) and Outpatient cohort (OC). The average z-score using ten-fold cross-validation is given for the trajectories of the SCC over days (last column). *ADL* Activities of Daily Living, *IV* Intravenous, *TPN* Total Parenteral Nutrition, *BP* Blood Pressure, *GGT* Gamma-Glutamyl transferase, *ULN* Upper Limit of Normal,

^a^Grouped as ‘infectious SCC’; […] left out for visualization.

## Discussion

Our results show that wearable-based RPM combined with a deep neural network model enables the calculation of an SCC-Score that allows for detecting and predicting SCC in patients receiving intensive treatment for hematological malignancies. Prediction was possible up to 48 h before documented SCC are diagnosed clinically. This study can be regarded as a successful ‘proof of principle’ for wearable-based RPM during oncological treatment where patients are at high risk for life-threatening complications.

Heterogeneous SCC in terms of type and severity were observed. As the trajectory of the SCC is diverse, the induced changes in vital signs and physical activity vary to a different degree. For example, an infection may develop over the course of hours and days, whereas a hypertensive crisis or cardiac arrhythmias can both occur and resolve from one moment to the other. The SCC-Scores represent this diversity.

The overall levels of the SCC-Scores observed for regular hours and non-regular hours in the two cohorts (IC vs OC) were different. The cause for this difference is not clear, it might reflect the higher physical activity levels of patients in the OC. Relative recording times in both cohorts were comparable^16^. The degree of change in the SCC-Score induced by SCC is similar in IC and OC, which results in a comparable AUROC analysis outcome (Table 2). Given the different levels in SCC-Scores between the cohorts implicates necessity to record data in a precise clinical context^17^. A solution to this problem may be on the one hand to collect a large amount of ‘regular’ data in patient populations with heterogenuous behaviour in focus. On the other hand our algorithm combines data from the individual patient with the complete body of data acquired from patients in a similar clinical situation. In combination these options may enable the ‘phenotyping’ of vital signs and physical activity measures to optimize the reference set and improve generalizability of this approach to new patients.

**Table 2:** SCC-Score performance.

Approach	Type	Model	regular hours [n]	non-regular hours [n]	SCC-Score regular hours [mean ± SD]	SCC-Score non-regular hours [mean ± SD]	*P*-value	Sensitivity/Specificity [%]	~95% Sensitivity/Specificity [%]	AUROC ( ± SD)
Patient non-specific	SCC	IC	1299	10,073	0.098 ± 0.037	0.144 ± 0.049	<0.0001	73.6/66.7	95.3/22.2	0.77 ± 0.02
		OC	526	1701	0.089 ± 0.045	0.132 ± 0.061	<0.0001	75.3/66.7	95.2/22.2	0.78 ± 0.02
		Total	1825	11,738	0.096 ± 0.038	0.146 ± 0.051	<0.0001	71.4/73.7	95.1/28.3	0.80 ± 0.01
	infectious SCC	IC	1486	8207	0.097 ± 0.038	0.145 ± 0.050	<0.0001	69.5/74.7	95.3/27.3	0.80 ± 0.01
		OC	541	1553	0.079 ± 0.039	0.122 ± 0.062	<0.0001	57.7/84.8	95.1/21.2	0.79 ± 0.03
		Total	2027	9760	0.097 ± 0.038	0.150 ± 0.051	<0.0001	74.6/72.7	95.2/31.3	0.82 ± 0.01
Patient-specific	SCC	IC	1299	10,073	0.112 ± 0.052	0.275 ± 0.132	<0.0001	79.7/87.9	95.2/56.6	0.91 ± 0.01
		OC	526	1701	0.103 ± 0.058	0.209 ± 0.113	<0.0001	77.4/81.8	95.2/41.4	0.87 ± 0.02
		Total	1825	11,738	0.111 ± 0.056	0.299 ± 0.141	<0.0001	81.6/88.9	95.2/61.6	0.93 ± 0.01
	infectious SCC	IC	1486	8207	0.111 ± 0.053	0.283 ± 0.138	<0.0001	82.5/87.9	95.0/62.6	0.93 ± 0.01
		OC	541	1553	0.091 ± 0.051	0.199 ± 0.114	<0.0001	71.9/88.9	95.1/37.4	0.88 ± 0.02
		Total	2027	9760	0.112 ± 0.055	0.304 ± 0.144	<0.0001	84.4/88.9	95.0/66.7	0.94 ± 0.01

SCC-Scores based on the patient’s non-specific and patient-specific approach of ‘hours for testing’ containing regular hours and non-regular hours are reported. These hours were previously unseen by the deep learning model. Differences in mean SCC-Scores in the respective cohorts (SCC_IC_, SCC_OC_, SCC_Total_) between regular hours and non-regular hours and respective *p*-values (two-sided *t*-test) are reported. To account for multiple testing, Bonferroni correction was applied and the significance level was set to 0.05/12 = 0.0042. Performance indicators (at Youden Index) of the SCC-Score were calculated for detection of SCC, and infectious SCC in patients in the IC, OC, and Total, separated for hours. In addition, specificity is reported at a sensitivity of ~95% to ensure a high ratio of SCC detection. AUROC of the SCC-Scores are given in the last column (standard deviation (SD) from ten-fold cross-validation).

In the subgroup analysis for infectious SCC, a transient increase of SCC-Scores before clinical SCC diagnosis (at *t* = 0 h) allows for the prediction of infectious SCC at an early stage. The SCC-Score shows a steeper slope before the diagnosis than the decrease in the hours post-diagnosis (Fig. 3). This increase could be driven by the uninhibited evolvement of e.g. an infection, whereas the decline is probably associated with the therapeutic intervention initiated. This phenomenon allows for the speculation that treatment success of an SCC or failure may also be tracked by RPM.

To detect the specific signatures in the recorded vital signs and physical activity induced by SCC, regular and non-regular hours during treatment were compared. In contrast to other studies, pre-treatment recordings were omitted, as it can be proposed that vital signs and physical activity differ significantly between pre-treatment and during treatment, even in the absence of SCC^18^.

The performance of the patient-specific analysis of our approach depends on the number of regular hours recorded for a single patient. If the number of recorded regular hours is low and does not represent a good approximation for the distribution of regular hours for a given patient, the false positive rate increases. False positives arise if for a regular hour in the test set no similar hour in the reference set can be found and consequently, this test hour is classified as SCC. It should be emphasized that a test hour and its best match in the reference set are typically found next to each other on the timeline (Supplementary Fig. 4a, b). This observation reflects the fact that the recorded time series of vital signs and physical activity are far from ergodic. Ergodicity implies that for each regular hour in the dataset, there exists another regular hour with similar features but sufficiently separated in time such that all time correlations are decayed. Therefore, the reported AUROC values of this work are upper bounds and can only be achieved in clinical practice for sufficiently long recordings of regular hours.

Training deep learning models on complex data follows the maxim that ‘big is better’, which refers to jointly enlarging models, data sets, and training times^19^. This study confirms this trend by taking training sets of different sizes but keeping model and training times constant (Supplementary Fig. 5). The employed strategy of training a single deep neural network to extract relevant features from raw data (end-to-end training) has the advantage that it can handle artefacts and data gaps, without the need of additional data pre-processing. Using a dilated residual network architecture as feature encoder, which has equivariance to time shifts as inductive bias, has the advantage to be more data efficient in comparison to other architectures, such as transformers^20^.

Patients’ responses of vital signs and physical activities to SCC of any kind can vary strongly. Therefore, we applied patient-specific evaluation instead of using rigid thresholds that apply to all patients. However, the patient-specific evaluation uses a single SCC-Score model trained on the totality of provided vital signs and physical activity measures from all patients. For real-world adaption, the trained deep learning model can be implemented on a smartphone, as the computationally demanding training of the model can be done remotely.

From a clinical point of view, it is desirable to minimize the risk of missing SCC. This choice is somewhat arbitrary and needs to be discussed according to the clinical context^18^. Depending on the situation under consideration and prior knowledge (e.g. given by a pre-test probability), clinicians can individually choose the decision boundary such that a certain balance of sensitivity and specificity is achieved. This decision boundary, which is directly related to the significance level of the statistical test, may also be adapted during real-world application when more information becomes available^21^. In general, the SCC-Score calculated by our model represents a single value that can be translated into actionable clinical information.

In the future, automated SCC detection by a wearable-based RPM in clinical oncology offers the option of permanent patient surveillance and may thereby improve complication management. Ideally, recorded data would be analyzed in real-time to provide actionable information for early and effective treatment. This may improve clinical pathways, e.g. implementation of demand-driven visits, which could reduce physicians‘ and nurses‘ workload in specialized clinics^22^. Furthermore, a decrease in the frequency of blood sampling during treatment of patients for their hematological malignancy is possible as recent research indicated a good correlation of wearable recorded vital signs with laboratory measurement results^23^. This approach may reduce treatment and disease burden by enabling optimal timing of interventions to counter SCC.

The sample size evaluated in this exploratory study is limited; however, this is the largest trial employing wearable-based RPM in patients treated for hematological malignancies^10^. Limitations of the wearable used in this study are described elsewhere^16^. Grading of SCC with Common Terminology Criteria for Adverse Events (CTCAE) grade ≥3 may influence vital signs and physical activity differently. Using this grading threshold for SCC omits lower grade complications, which, however, may already be of therapeutic relevance and affect the patient’s wellbeing. Not all SCC may affect vital signs and physical activity to the degree that they are likely to be detected by a wearable-based RPM approach; infection-induced SCC might lead to a stronger’signal’ than some other SCC and may therefore be an ideal target for RPM. However, it is unclear which sets of parameters are required for optimal SCC detection. This question must be addressed in subsequent evaluations.

In summary, this study provides proof of the principle that SCC in a vulnerable patient population of patients receiving treatment for hematological malignancies can be detected and predicted with an innovative approach, based on continuously recorded wearable data combined with a self-supervised deep learning model. Prospective confirmatory studies are needed to document the clinical benefit of this approach in clinical practice.

## Methods

### Study design and setting

This was an open-label, single-arm, single-center, investigator-initiated cohort study covering patients with a hematological malignancy receiving oncological treatment (chemotherapy alone or in combination with radiotherapy and/or hematopoietic stem cell transplantation) (Supplementary Fig. 1). The study was conducted at the Department of Hematology, Oncology, and Clinical Immunology of the University Hospital Düsseldorf, Germany^16^. The study was approved by the Ethics Committee of the Medical Faculty of the Heinrich Heine University Düsseldorf and was registered in the German clinical trials register (DRKS00014782) on 29 May 2018. Before study participation, patients were informed that they would not derive immediate individual benefits from study participation. All patients provided written informed consent before study inclusion.

### Participants

Inclusion criteria were patients’ age ≥18 years and an indication for a treatment protocol with expected hematotoxicity according to CTCAE grade 4 alone or in combination with stem cell transplantation. Exclusion criteria were medical or mental conditions impairing the ability to continuously wear the wearable (e.g. dementia, skin abnormalities) and active implants, which might impair recordings. During visits, the following data were obtained: medical history, comorbidities, symptoms, physiological parameters, laboratory values, and physical examination. A convenience sample of 79 patients was recruited: 54 patients were treated in the hospital (inpatient cohort (IC)), and 25 patients received outpatient-based treatment (outpatient cohort (OC)) (Supplementary Table 1, 2).

Patients and clinical staff were blinded for wearable data.

### Data collection and preparation

The commercially available wearable (Everion, Biovotion AG, Switzerland) employed is a CE-marked medium-risk device (class IIa) according to Directive 93/42/EEC (firmware used was for clinical investigation only). Different sensors implemented in this wearable were used for non-invasive monitoring of vital signs and physical activity (e.g. photoplethysmography, temperature probe, accelerometer). Longitudinally recorded parameters, such as heart rate, temperature, respiratory rate, and physical activity, and if applicable, respective quality indices were calculated with proprietary methods implemented in the firmware (Supplementary Table 3). Raw signals were acquired with a frequency of >30 Hz; calculated parameters were stored with a rate of 1 Hz, resulting in up to 3,600 data points per hour. The battery of the wearable had to be recharged daily for 90 min.

Two wearables for alternate use were assigned to each patient at the baseline visit before starting treatment to enable continuous wearable-based monitoring of vital signs and physical activity in these patients. The frequency of subsequent study visits (app. every 90 h for device swap) was determined by the limited data storage capacity of the wearable.

Non-hematological SCC were defined by meeting the criteria of CTCAE (v4.03) grade ≥3^24^. Clinical documentation (visit entries, laboratory results, diagnostic results) was independently and retrospectively reviewed by two investigators (PR, MJ) for the occurrence of SCC. For each clinically documented SCC, a starting time point was noted. Infectious SCC with no focus of origin were classified as ‘Infections and infestations—other’. Recovery from a SCC was defined as the absence of documented clinical symptoms, pathological laboratory, and diagnostic results to consider varying trajectories of different types of SCC, e.g. a hypertensive crisis with rapid onset compared to an infection, which develops over several hours/days.

**Fig. 4:**
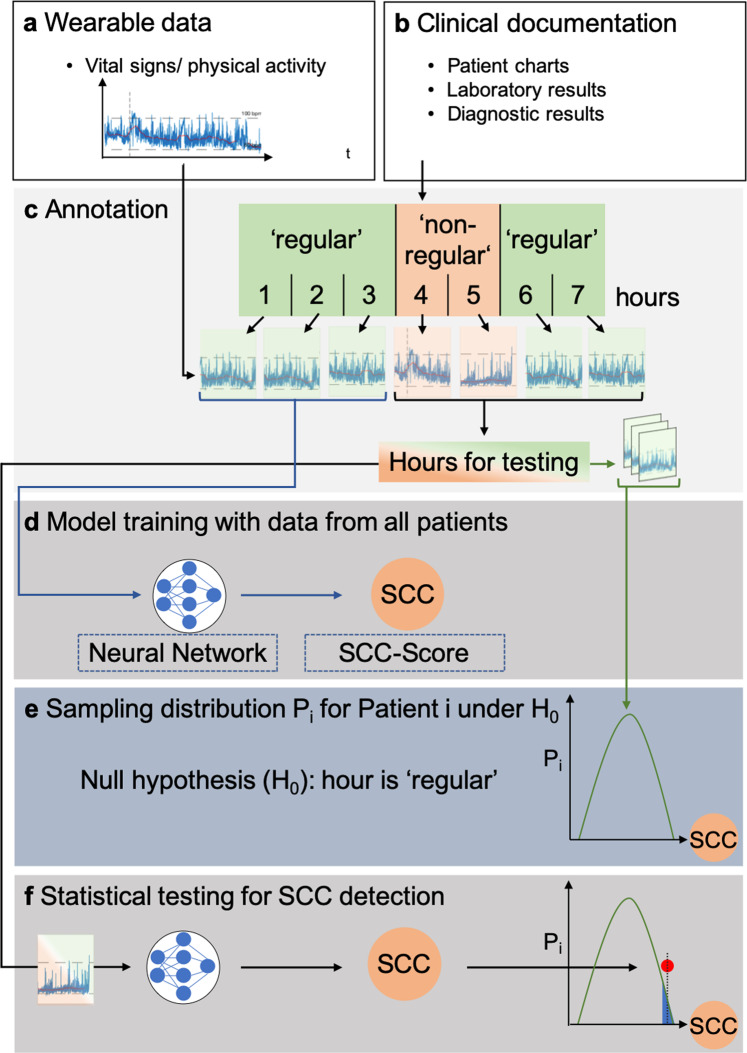
Development of a deep learning model for calculation of an SCC-Score. **a** Time series of vital signs and physical activity recorded by a medical wearable. **b** Clinical documentation, such as patient charts or laboratory results, that were reviewed for identifying SCC events. **c** According to the clinical documentation, the hours without evidence of SCC were annotated as regular hours, the remaining hours were regarded as non-regular. **d** regular hours for each individual patient were randomly split into two datasets: 90% for training and 10% for testing and generating a null-distribution. For cross-validation, the splitting was repeated ten times. For training the deep learning model, the regular hours were presented to a deep neural network as part of a self-supervised contrastive learning objective. An SCC-Score based on the similarity between a test hour and the closest regular hour from the training set was calculated. **e** A null-distribution of SCC-Scores from regular hours in the test set was established. **f** For a given hour, a statistical test under the null-distribution was applied to detect SCC, with a significance level selected by clinical requirements.

Time series data were recorded for IC and OC patients that together formed the total cohort (Fig. 4). Data sets were split into hours according to their timestamps, and only hours with ≥3000 data points were included to ensure sufficient information content among hours (Supplementary Fig. 2a). No predefined quality constraints were used. For each day with documented SCC, all 24 h were annotated as *non-regular*, regardless of the exact timestamp of the respective SCC start. Infectious SCC received special annotation for later subgroup analyses. Since changes in vital signs and physical activity may already occur before SCC criteria are fulfilled, a time buffer was introduced given by 48 h before the day with a timestamp of SCC onset and 24 h post-recovery from an SCC. The resulting periods were also annotated as *non-regular*. Hours outside the non-regular hours were annotated as *regular* (Supplementary Fig. 2b).

A total of 140 SCC events were extracted from the clinical documentation of the patients (Table 1). The data of two patients without regular hours and early study withdrawal were excluded. The cumulative incidence of SCC in the IC was 90.7%, and those in the OC were 48.0%. More than one SCC occurred in 30 patients in the IC and 3 patients in the OC. Infectious SCC accounted for 65.0% of the total SCC and were the most frequent SCC in both cohorts (IC 63.7%, OC 75.0%).

Wearable data were recorded for 24,047 h for the IC patients; the median recording time per patient was 457.4 (IQR 324.3–538.5) hours. The OC patients had 7187 h of total recording time, with a median of 315.5 (227.4–340.8) hours per patient. Hours meeting data constraints were 23,262 h (96.4%) in the IC and 6955 h (96.3%) in the OC.

### Deep learning model

For classifying the recorded hours, a self-supervised contrastive learning method was used that learns to organize complex structured data such that data points with similar features are located close to each other. In particular, a ResNet architecture composed of 24 residual blocks was employed, followed by a linear neural network with 128 output nodes as feature extractor. The complete network was trained end-to-end, using a temperature scaled cross entropy loss. The contrastive learning objective enforces the normalized, 128-dimensional feature vectors to be aligned for adjacent time intervals and disaligned for temporally distant time intervals^25^. To classify a given test hour as regular or SCC event, the similarity between features of the test hour and the annotated regular hours, which represent the training set are computed. Test hours with low similarity were treated as anomalies. Only features with a high signal-to-noise ratio across all patients were considered, such that the model can be applied to new patients without retraining. These robust features were selected by enforcing the model to be invariant against random shifts of the time frame by less than half an hour. The training dataset was generated by randomly collecting 90% of the regular hours for each patient^26^. The remaining 10% of the regular and non-regular hours were used for testing. After training, the features extracted by the model can be used to identify deviations from the regular hours to detect SCC.

To quantify anomalies, the extracted features for each hour of vital signs and physical activity were represented as a high dimensional vector of unit length. For each hour of the test set, a corresponding *reference set* was defined. The reference set either represented the complete training set that included all patients (patient-non-specific approach) or just the regular hours of the training set that belonged to the same patient as the corresponding test hour (patient-specific approach). The similarity between different hours can be quantified by computing the scalar product between feature vectors (cosine similarity^25^). To evaluate a test hour, an SCC-Score was defined as one minus the maximum of all cosine similarities between the test hour and the hours of the reference set. A higher SCC-Score indicated a larger deviation from what is expected to be a regular hour. The SCC-Scores for the regular hours of the test set represent the null-distribution. The null hypothesis assumes an hour to be regular and was rejected for any hour with SCC-Score above a pre-specified significance level (Fig. 4). The significance level has to be pre-specified to meet clinical requirements and can be interpreted as the decision boundary for the SCC classification problem. A patient-specific evaluation can be realized by restricting the reference set and the null-distribution to the regular hours of a single test patient. This patient-specific restriction is used for statistical testing but not for training the score, as the score is always trained on the regular hours of the total cohort. The SCC-Scores of the test set were evaluated per hour, even though SCC events were annotated per day (24 h). Averages of SCC-scores over 24 h were denoted as per*-*day SCC-scores.

### Anomaly detection method

Identifying anomalies is inherently a highly unbalanced binary classification problem, where normal or typical data points are highly abundant and abnormal data points or outliers are typically rare. The distribution of possible anomalies (out-distribution) is unknown but assumed to be much broader than the distribution of normal data points (in-distribution)^27^. To detect anomalies, we follow the strategy of finding in-distribution specific features, where we assume the existence of sufficiently large subsets of data points that share at least some of these features. This strategy implies that normal data points typically show high proximity to at least one of the subsets in feature space, whereas outliers are expected to be located more distant^14^.

### Learning in-distribution specific features

One way to learn in-distribution specific features is to augment the dataset with examples that show high variance for features that are not in-distribution specific and are expected to co-occur also in outliers but little variance for in-distribution specific features. For instance, given an in-distribution that consists of images of natural objects (e.g. images of ‘cat’, ‘ship’), then transformations applied to each image, such as combinations of moderate cropping and resizing, moderate color jitter, and horizontal flip, have a strong effect on individual pixel values (low-level features) but little effect on the object category (high-level features)—a ‘cat’ remains a ‘cat’. Consequently, the information shared between any two transformations of the same image (positive pair) can be used to define the in-distribution specific features. The downside of this approach is that we must know a priori which transformations can significantly shift data points but leave in-distribution specific features invariant.

For the time series data used in this work we don’t generate new data but define as positive pair any two-time intervals, $x$ and *x*′ of 1000 s length that were randomly selected within the same hour but separated by at least 500 s. The valid transformations are random shifts of these intervals within a given hour by at most 500 s. To extract the features that are invariant under these transformations, we map each time interval, $x$, to a $d$ dimensional feature vector $h$, with the help of a deep convolutional neural network, $h={f}_{0}(x)$, as a feature extractor. The network ${f}_{0}(x)$ is trained by a Self-Supervised Contrastive Learning objective, which approximately maximizes the mutual information for the sampled positive pairs across all recorded hours.

### Self-supervised contrastive learning

The self-supervised contrastive learning objective aligns feature vectors that share invariant information in feature space (positive pairs) and simultaneously pushes feature vectors apart that don’t share invariant information (negative pairs). Negative pairs are not generated explicitly but arise from building pairs of time intervals from different hours. Let ${h}_{i}={f}_{0}({x}_{i})$ and ${h}_{i}^{{\prime} }={f}_{0}({x}_{i}^{{\prime} })$ be feature vectors for two randomly selected time intervals within the same hour $i$ of the training dataset. Then ${(x}_{i},{x}_{i}^{{\prime} })$ is a positive pair and ${(x}_{i},{x}_{k}^{{\prime} })$ a negative pair for $i\ne k$. We define the similarity between feature vectors by ${{sim}(h}_{1},{h}_{2}):=\frac{{h}_{1}^{T}{h}_{2}}{\parallel {h}_{1}\parallel {\parallel h}_{2}\parallel }$, which is the dot product between ${l}_{2}$ -normalized feature vectors ${h}_{1}$ and ${h}_{2}$ (cosine similarity). In self-supervised contrastive representation learning, a loss function can be defined by^25^

1 $${{\mathcal{L}}}_{{i}}=-\log \frac{{\rm{exp }}({sim}({h}_{i},{h}_{i}^{{\prime} })/\tau )}{{\rm{exp }}({sim}({h}_{i},{h}_{i}^{{\prime} })/\tau )+{\sum }_{k\ne i}^{n}\left[{\rm{exp }}({sim}({h}_{i},{h}_{k}^{{\prime} })/\tau )+{\rm{exp }}({sim}({h}_{i}^{{\prime} }{,h}_{k})/\tau )\right]}$$

where $\tau > 0$ is a scalar temperature parameter, $n$ is the number of randomly selected hours (minibatch size), with two randomly selected 1000 s intervals, ${x}_{i}$ and ${x}_{i}^{{\prime} }$, per hour. The temperature parameter was set to $\,\tau$ = 0.07. The neural network ${f}_{0}$ was realised by a ResNet architecture. The ResNet architecture is composed of 24 residual blocks. Each residual block consists of two convolutional layers, each followed by batch normalisation and ReLU activation. The convolutional layers have filters of size 16 with stride 2. Each convolutional layer has 32 filters which doubles every 12 blocks. The Resnet Output is passed into a projection head consisting BN, ReLU and a linear layer. Details of the architecture can be found in Shenda et al.^26^. The encoder, ${f}_{0}$ , maps inputs to a 128-dimensional feature space embedding. The outputs of this network are ${l}_{2}$-normalized, $h/\parallel h\parallel$, and consequently mapped onto a unit hypersphere. Five of the 12 wearable signals of vital signs and physical activity come with a quality index that ranges from 0–100. Every second of the five signals with quality index are shown at their corresponding position in the 101-dimensional quality index vector. The remaining entries of this vector are set to zero. This representation results in a $5\times 101+7=512$ dimensional input for every second. An Adam optimizer with parameters ${\beta }_{1}$ = 0.9, ${\beta }_{2}$ = 0.98, initial learning rate of ${10}^{-3},$ and weight decay of ${10}^{-3}$ was used. The model was trained with batch size 128 for 500 epochs.

### Data preprocessing

The input features were stored with sample rate of 1 Hz by the wearable device. The dataset of vital signs and activity data is represented as ${\left\{{X}_{n}\right\}}_{n=1}^{N}$, with ${X}_{n}\in {{\mathbb{R}}}^{{DxT}}$, where $N$ is the number of hours across all patients, $D$ is the input dimension and $T$ is the number of consecutive time points within 1 h. We take $T$ = 3000, which is less than the expected $T$ = 3600 s for an hour, as we frequently observed interruptions and therefore shorted $T$ to keep most of the consecutive time series in the data.

### Score function for SCC detection

From the set of feature vectors for the training examples, ${{\mathcal{D}}}_{{train}}={\left\{{h}_{m}\right\}}_{m=1}^{{KN}}$, with $K$ the number of randomly selected 1000 s intervals per hour, a score function can be defined to evaluate whether a given test sample should be classified as outlier (SCC). For a given feature vector of a test example, ${h}_{{test}}={f}_{0}({x}_{{test}})$, the cosine similarity to the nearest training example in ${{\mathcal{D}}}_{{train}}$ is taken as a score for detecting SCC samples. Our cosine similarity-based SCC score, ${S}_{{SCC}}(x)$, is defined as

2 $${S}_{{SCC}}\left({{{x}}}_{{test}}\right)\;{\rm{:=}}\;\frac{1}{K}\mathop{\sum }\limits_{k=1}^{K}\left[1-\mathop{{\rm{max }}}\limits_{{h}_{m}\in {{\mathcal{D}}}_{{train}}}{sim}({h}_{m},{h}_{{test}}^{k})\right]$$

We take $K$ = 6 and the corresponding ${set}\{h_{test}^{k}\}$ are random samples from the same hour. The test example, ${{{x}}}_{{test}}$, is classified as SCC if the SCC score is above a threshold. For patient-specific evaluation, the cosine similarity is calculated with respect to the nearest example among the training regular hours of the patient being tested rather than the training regular hours of all patients.

### Statistical analysis

Primary outcomes were the detection and prediction of clinically documented SCC by the SCC-Score. Subgroup analysis was evaluated for infectious SCC. For statistical analysis, differences between means of hours annotated as regular and non-regular obtained from SCC_IC_-, SCC_OC_- and SCC_Total_-score were tested for significance using a two-sided *t*-test, and adjustment for multiple comparisons was performed by using Bonferroni correction. For clinical requirements, specificity was reported at a sensitivity of ~95%.

To address overfitting, a ten-fold cross-validation for the 90/10 split of regular hours was carried out, which included retraining the model. Statistical significance was tested by an ANOVA between the cross-validation splits of regular and non-regular hours (Supplementary Fig. 3). Receiver Operating Characteristics (ROC) analysis was carried out and the area under the ROC curve (AUROC) was computed to evaluate the performance of the different approaches in different settings. Standard errors of the AUROC curves were computed from ten-fold cross-validation in combination with bootstrapping over the test set. The null distribution was generated by computing SCC-Scores for the regular hours of the reference set, using cross-validation. To assess SCC-Score prediction capabilities for infectious SCC (Table 1), the performance of the score in the 120 h before and after the time stamp of diagnosis (*t* = 0 h) were analyzed. An average *z*-score of ten-fold cross-validation was calculated to assess the relative effect of a SCC on the SCC-Score. A *p*-value < 0.05 was considered significant. For data and statistical analysis, open-source software tools were used.

### Reporting summary

Further information on research design is available in the Nature Research Reporting Summary linked to this article.

## Supplementary information

Reporting Summary

Supplementary Material

## Data Availability

The datasets generated during and/or analysed during the trial are available from the corresponding author on reasonable request.

## Code availability

Scripts for data analysis are available at https://github.com/HHU-MMBS/ASSISTO-Project.
