# Supplementary material for: Wearable based monitoring and self-supervised contrastive learning detect clinical complications during treatment of Hematologic malignancies
Source: NPJ Digit Med. 2023 Jun 2;6:105. doi: 10.1038/s41746-023-00847-2 (PMC10238496; doi:10.1038/s41746-023-00847-2)
Supplement: Supplementary file 1 — Reporting Summary [file 41746_2023_847_MOESM1_ESM.pdf]

nature portfolio

Corresponding author(s): Dr. Malte Jacobsen

Last updated by author(s): 03 May 2023

Reporting Summary

Nature Portfolio wishes to improve the reproducibility of the work that we publish. This form provides structure for consistency and transparency in reporting. For further information on Nature Portfolio policies, see our Editorial Policies and the Editorial Policy Checklist.

Statistics

For all statistical analyses, confirm that the following items are present in the figure legend, table legend, main text, or Methods section.

n/a	Confirmed	
☐	☒	The exact sample size (*n*) for each experimental group/condition, given as a discrete number and unit of measurement
☐	☒	A statement on whether measurements were taken from distinct samples or whether the same sample was measured repeatedly
☐	☒	The statistical test(s) used AND whether they are one- or two-sided<br>*Only common tests should be described solely by name; describe more complex techniques in the Methods section.*
☒	☐	A description of all covariates tested
☐	☒	A description of any assumptions or corrections, such as tests of normality and adjustment for multiple comparisons
☐	☒	A full description of the statistical parameters including central tendency (e.g. means) or other basic estimates (e.g. regression coefficient) AND variation (e.g. standard deviation) or associated estimates of uncertainty (e.g. confidence intervals)
☐	☒	For null hypothesis testing, the test statistic (e.g. *F*, *t*, *r*) with confidence intervals, effect sizes, degrees of freedom and *P* value noted<br>*Give P values as exact values whenever suitable.*
☒	☐	For Bayesian analysis, information on the choice of priors and Markov chain Monte Carlo settings
☒	☐	For hierarchical and complex designs, identification of the appropriate level for tests and full reporting of outcomes
☒	☐	Estimates of effect sizes (e.g. Cohen's *d*, Pearson's *r*), indicating how they were calculated

Our web collection on statistics for biologists contains articles on many of the points above.

Software and code

Policy information about availability of computer code

Data collection	For downloading vital signs and physical activity data from the wearable, non-commercially available software from the wearable manufacturer was used.
Data analysis	For data and statistical analysis, open-source software tools were used (Python, version 3.6.5).<br>https://github.com/HHU-MMBS/ASSISTO-Project

For manuscripts utilizing custom algorithms or software that are central to the research but not yet described in published literature, software must be made available to editors and reviewers. We strongly encourage code deposition in a community repository (e.g. GitHub). See the Nature Portfolio guidelines for submitting code & software for further information.

Data

Policy information about availability of data

All manuscripts must include a data availability statement. This statement should provide the following information, where applicable:

- Accession codes, unique identifiers, or web links for publicly available datasets
- A description of any restrictions on data availability
- For clinical datasets or third party data, please ensure that the statement adheres to our policy

The datasets generated during and/or analysed during the trial are available from the corresponding author on reasonable request.

March 2021

Human research participants

Policy information about studies involving human research participants and Sex and Gender in Research.

Reporting on sex and gender	*Use the terms sex (biological attribute) and gender (shaped by social and cultural circumstances) carefully in order to avoid confusing both terms. Indicate if findings apply to only one sex or gender; describe whether sex and gender were considered in study design whether sex and/or gender was determined based on self-reporting or assigned and methods used. Provide in the source data disaggregated sex and gender data where this information has been collected, and consent has been obtained for sharing of individual-level data; provide overall numbers in this Reporting Summary. Please state if this information has not been collected. Report sex- and gender-based analyses where performed, justify reasons for lack of sex- and gender-based analysis.*
Population characteristics	A total of 79 patients participated in the study: 54 inpatients (44% female, age median [IQR] 56 [49-62]) and 25 outpatients (44% female, age 54 [50-62]).
Recruitment	Patients receiving oncological treatment at the Department of Hematology, Oncology, and Clinical Immunology of the University Hospital Duesseldorf, Germany were consecutively screened for their eligibility to participate in the study.
Ethics oversight	The trial was approved by the Ethical Committee of the University Hospital Duesseldorf, Germany

Note that full information on the approval of the study protocol must also be provided in the manuscript.

Field-specific reporting

Please select the one below that is the best fit for your research. If you are not sure, read the appropriate sections before making your selection.

☒ Life sciences ☐ Behavioural & social sciences ☐ Ecological, evolutionary & environmental sciences

For a reference copy of the document with all sections, see nature.com/documents/nr-reporting-summary-flat.pdf

Life sciences study design

All studies must disclose on these points even when the disclosure is negative.

Sample size	A convenience sample of 79 patients was recruited: 54 patients were treated in the hospital (inpatient cohort (IC)), and 25 patients received outpatient-based treatment (outpatient cohort (OC)).
Data exclusions	Hours of vital signs and physical activity data recorded by the wearable with less than 3,000 datapoints were excluded.
Replication	10-fold cross validation was performed to verify the reproducibility of the data. The reproducibility tests were successful with some variance (see Tab 2 in the manuscript).
Randomization	N/A
Blinding	N/A

Reporting for specific materials, systems and methods

We require information from authors about some types of materials, experimental systems and methods used in many studies. Here, indicate whether each material, system or method listed is relevant to your study. If you are not sure if a list item applies to your research, read the appropriate section before selecting a response.

Materials & experimental systems

n/a	Involved in the study	
☒	☐	Antibodies
☒	☐	Eukaryotic cell lines
☒	☐	Palaeontology and archaeology
☒	☐	Animals and other organisms
☐	☒	Clinical data
☒	☐	Dual use research of concern

Methods

n/a	Involved in the study	
☒	☐	ChIP-seq
☒	☐	Flow cytometry
☒	☐	MRI-based neuroimaging

Clinical data

Policy information about clinical studies

All manuscripts should comply with the ICMJE guidelines for publication of clinical research and a completed CONSORT checklist must be included with all submissions.

Clinical trial registration	DRKS00014782 German clinical trials register
Study protocol	The trial protocol is available from the corresponding author
Data collection	Recruitment occurred at the Department of Hematology, Oncology, and Clinical Immunology of the University Hospital Duesseldorf, Germany, between November 2018 and January 2020.
Outcomes	Primary outcomes were the detection and prediction of clinically documented SCC by the SCC-Score. Subgroup analysis was evaluated for infectious SCC. For statistical analysis, differences between means of hours annotated as regular and non-regular obtained from SCCIC-, SCCOC- and SCCTotal-Score were tested for significance using a two-sided t-test, and adjustment for multiple comparisons was performed by using Bonferroni correction.
