# Supplementary material for: Wearable based monitoring and self-supervised contrastive learning detect clinical complications during treatment of Hematologic malignancies
Source: NPJ Digit Med. 2023 Jun 2;6:105. doi: 10.1038/s41746-023-00847-2 (PMC10238496; doi:10.1038/s41746-023-00847-2)
Supplement: Supplementary file 2 — Supplementary Material [file 41746_2023_847_MOESM2_ESM.pdf]

Supplementary Material

Patient characteristics	Inpatient cohort (n=54) No. (%)	Outpatient cohort (n=25) No. (%)	Total No. (%)
Male<br>Female	30 (55.6)<br>24 (44.4)	14 (56.0)<br>11 (44.0)	44 (55.7)<br>35 (44.3)
Age [years] (median (IQR)	56 (49-62)	54 (50-62)	55 (50-62)
BMI [kg/m²]	25.0 (21.7-28.5)	24.9 (23.3-27.1)	25.0 (22.1-28.0)
Hematological malignancy			
Acute leukemia (ALL, AML)	21 (38.9)	12 (48.0)	33 (41.8)
MDS, MPN (PMF, CML)	20 (40.7)	0 (0.0)	21 (26.6)
Other (CLL, HL, NHL, Myeloma, Germ cell)	13 (20.4)	13 (52.0)	25 (31.6)
Comorbidities			
Arterial hypertension	13 (24.1)	14 (56.0)	27 (34.2)
Diabetes mellitus (Type 1 and 2)	2 (3.7)	0 (0.0)	2 (2.5)
Macrovascular event (e.g. Stroke)	1 (1.9)	1 (4.0)	2 (2.5)
Heart failure with reduced ejection fraction	0 (0.0)	0 (0.0)	0 (0.0)
Arrhythmias	2 (3.7)	2 (8.0)	4 (5.0)
Concomitant medication			
Antiplatelet drugs	3 (5.6)	7 (28.0)	10 (12.7)
Beta-blocker	5 (9.3)	5 (20.0)	10 (12.7)
Calcium channel blocker	5 (9.3)	11 (44.0)	16 (20.0)
Renin-angiotensin system inhibitors	9 (16.7)	7 (28.0)	16 (20.0)
Other antihypertensives	4 (7.4)	0 (0.0)	4 (5.0)

Supplementary Table 1

Patient characteristics, malignancies, comorbidities, and concomitant medication of the patients in the Inpatient and Outpatient cohort; BMI: Body Mass Index, ALL: Acute lymphocytic leukemia, AML: Acute myeloid leukemia, MDS: Myelodysplastic syndrome, MPN: Myeloproliferative neoplasms, PMF: primary myelofibrosis, CML: Chronic myeloid leukemia, CLL: Chronic lymphocytic leukemia, HL: Hodgkin lymphoma, NHL: Non-Hodgkin lymphoma

a	Inpatient cohort	No. (%)
	Allogenic stem cell transplantation	48 (88.9)
	Autologous stem cell transplantation	6 (11.1)
	Conditioning protocol	
	Alkylator based (Treosulfan, Busulfan, Melphalan)	29 (53.7)
	FLAMSA-based	17 (31.5)
	TBI-based	7 (13.0)
	Other	1 (1.9)
	Intensity of conditioning regimens	
	Reduced intensity regiments	30 (55.6)
	Myeloablative conditioning	24 (44.4)
	GvHD prophylaxis	
	Antithymocyte globulin (ATG)	33 (61.1)
**b**	Outpatient cohort	No. (%)
	High-/Intermediate-dose Cytarabine with or without Mitoxantron	14 (56.0)
	High-dose Cyclophosphamide	9 (36.0)
	Others (e.g. Ifosfamide, Carboplatin, Etoposide)	2 (8.0)

Supplementary Table 2

The treatment regimen for Inpatient (a) and Outpatient cohort (b).

Parameter	Unit	Quality Index
Heart rate	30 – 240 beats per minute	X
Oxygen saturation	65 – 100%	X
Perfusion index	0 – 255 (arbitrary)	
Activity classification	Categorical	X*
Activity	0 – 255 (arbitrary)	
Steps	0 – 65,535 per day	
Blood pressure wave	0 – 5.1 (arbitrary)	
Heart rate variability	0 – 255 ms (RMSSD)	X
Respiration rate	6 – 30 per minute	X
Energy expenditure	0 – 65,535 kcal per day	X
Temperature	0 – 60°C	
Inter-beat interval	1 – 4,095 ms	X*
Electrodermal activity	0 – 21.8 kOhm	

Supplementary Table 3

Vital signs and physical activity parameters are provided by the medical wearable according to the instruction for use. RMSSD – root mean square of successive differences between normal heartbeats; *not used for the calculations

Hours before SCC-diagnosis	Model	regular hours [n]	non-regular hours [n]	SCC-Score regular hours [mean±SD]	SCC-Score non-regular hours [mean±SD]	P-Value	Sensitivity / Specificity [%]	AUROC (±SD)
-24	IC	698	60	0.096±0.011	0.144±0.012	<0.0001*	76.7/87.8	0.89±0.01
	OC	245	12	0.078±0.021	0.121±0.024	0.001*	81.0/73.5	0.83±0.03
	Total	943	72	0.097±0.007	0.149±0.010	<0.0001*	81.2/85.7	0.90±0.01
-48	IC	696	56	0.096±0.011	0.140±0.012	<0.0001*	77.9/81.6	0.86±0.01
	OC	245	10	0.078±0.020	0.113±0.023	0.004*	64.1/89.8	0.81±0.03
	Total	941	66	0.097±0.007	0.143±0.001	<0.0001*	73.9/87.8	0.88±0.01
-72	IC	664	39	0.097±0.011	0.106±0.012	0.088	81.9/30.6	057±0.05
	OC	238	8	0.078±0.021	0.082±0.020	0.653	61.6/55.1	0.58±0.07
	Total	902	47	0.096±0.007	0.105±0.001	0.044	62.8/46.9	0.56±0.03

Supplementary Table 4

Hours assessed by the respective model to estimate risk for infectious SCC in periods -72, -48, and -24 hours before a diagnosis was documented (0 hours) in patients in the IC, OC, and for both cohorts. Mean and standard deviation with respective models was calculated. Differences between regular and non-regular hours were tested using a two-sided t-test test. To account for multiple testing, Bonferroni correction was applied and the significance level was set to 0.05/9=0.0056. Significant p-values are marked with *. Sensitivity and Specificity at the Youden index are reported.

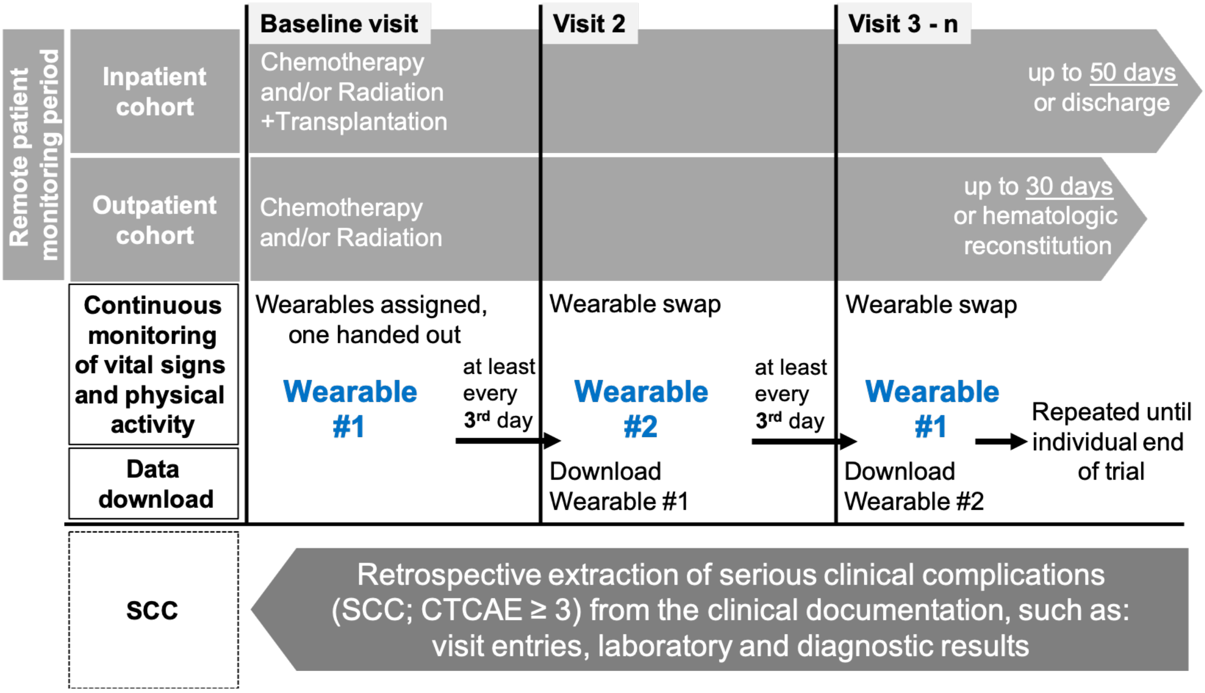

Supplementary Figure 1

Study design: Procedures applied to enable continuous wearable-based remote monitoring of vital signs and physical activity. Data were recorded in inpatients and outpatients allocated to two different cohorts. Patients attended the baseline visit before starting oncological treatment. At this visit, two wearables (#1 and #2) were assigned to each patient. At Visit 2, wearable #1 was swapped to #2, and data of #1 were downloaded. The frequency of subsequent visits (including swapping wearables) was determined by the limited data storage capacity of the wearable (no web application for data download was used due to regulatory requirements) to every 3rd day. The investigators retrospectively identified serious clinical complications from the clinical documentation.

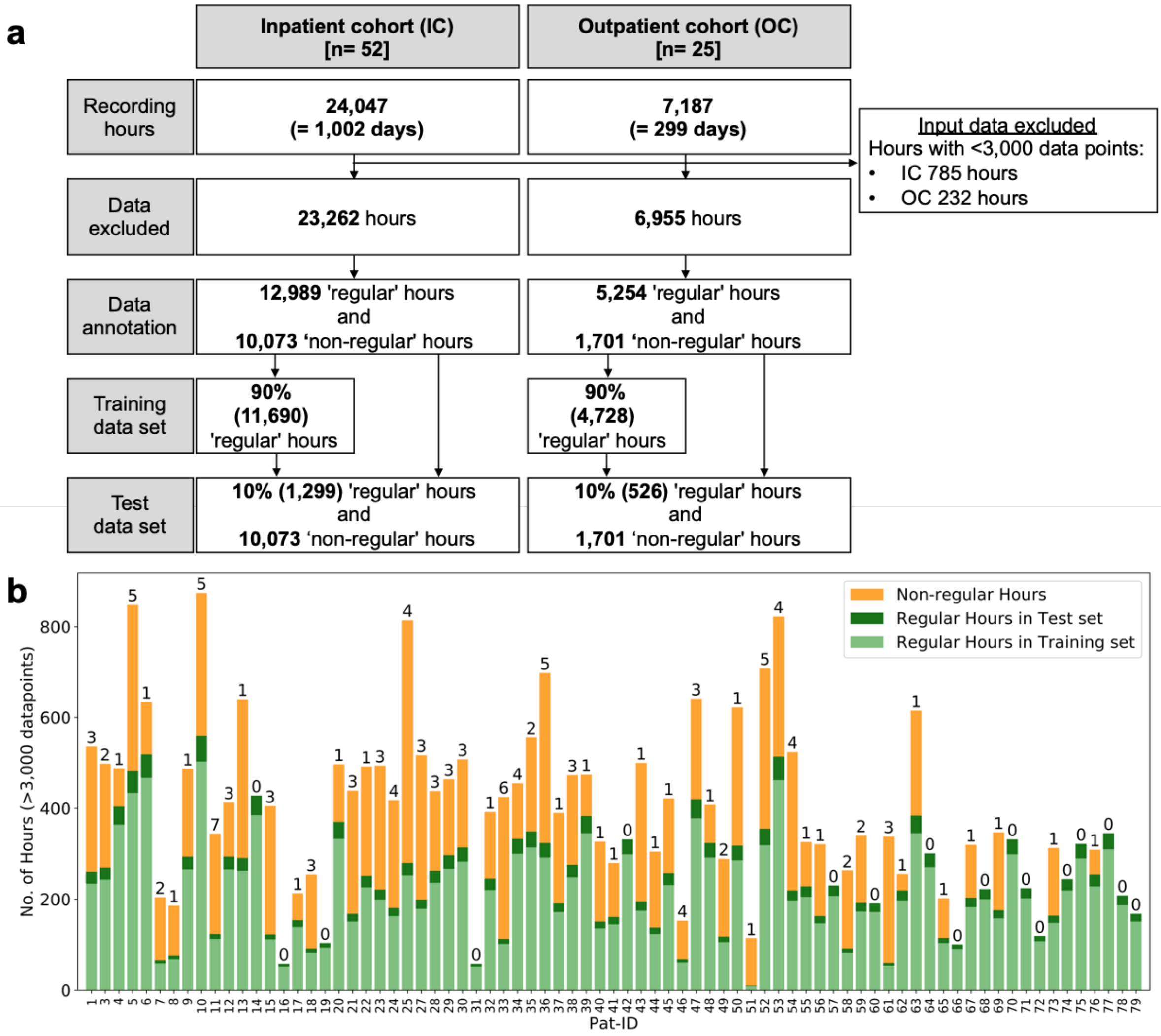

Supplementary Figure 2

(a) Acquisition of input data in the Inpatient cohort (IC) and Outpatient cohort (OC). For all patients only, hours were included if they had ≥3,000 data points per hour (of maximal 3,600). Data annotation refers to the separation of hours without serious clinical complications (SCC) (= regular hours) and non-regular hours. Data of two patients without recording hours annotated as regular hours were excluded. Allocation of data for training data set and test dataset: For training, the regular hour's dataset was randomly divided into two datasets (90% training data set, 10% test data set). This split was equivalently applied to the datasets of each patient.
(b) Number of hours per patiennt is given in numerical order of recruitment of the 79 patients (first 54 for IC and subsequent 25 for OC). For each patient, the orange bar indicates the amount of non-regular hours, whereas the dark green indicates regular hours in the test set and light green in the training set. The number over the bar represents the number of SCC that occurred in each patient.

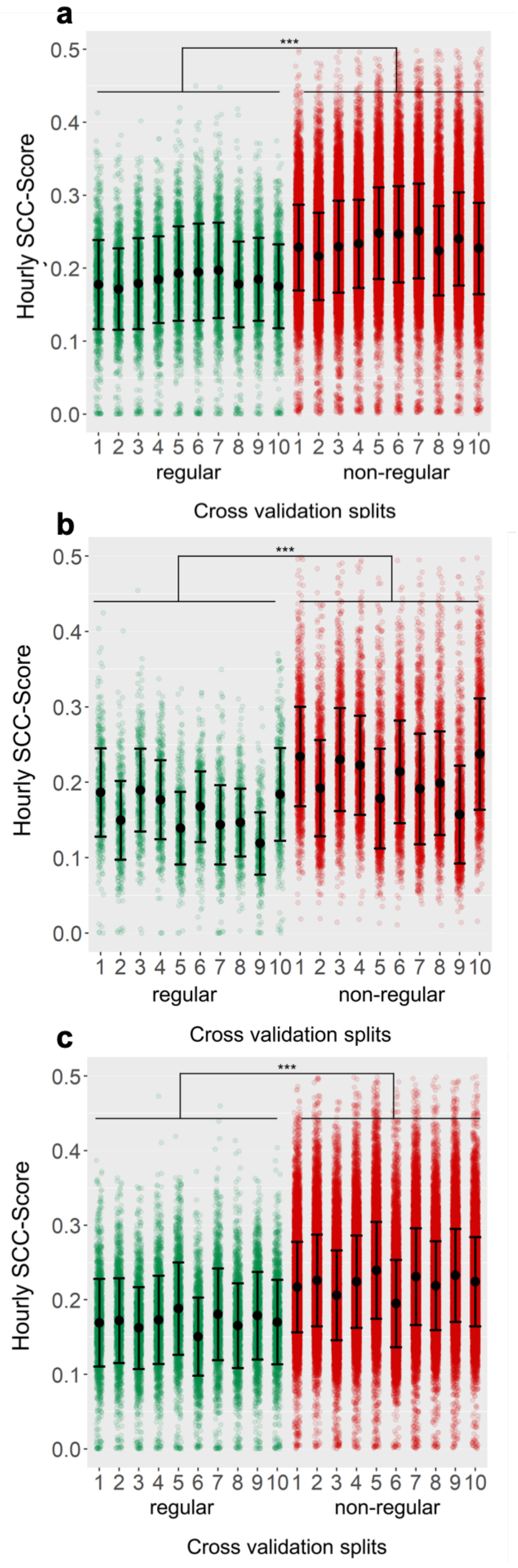

Supplementary Figure 3

Distribution of SCC-Scores calculated by the deep learning model for regular hours (green dots) and non-regular hours (red dots) for (a) Inpatient cohort (SCC_{IC}), (b) Outpatient cohort (SCC_{OC}), and (c.) Total cohort (SCC_{Total}) for ten different data splits (cross-validation). The black dot indicates the mean value of each data set. The black error bars indicate the standard deviation. Statistical significance was tested by an ANOVA between regular and non-regular hours and is indicated by asterisks (***$p<0.001$). In the post-hoc analysis considering per-fold comparisons all (two-sided) t-tests, applying Bonferroni corrections, remained significant ($p<0.001$).

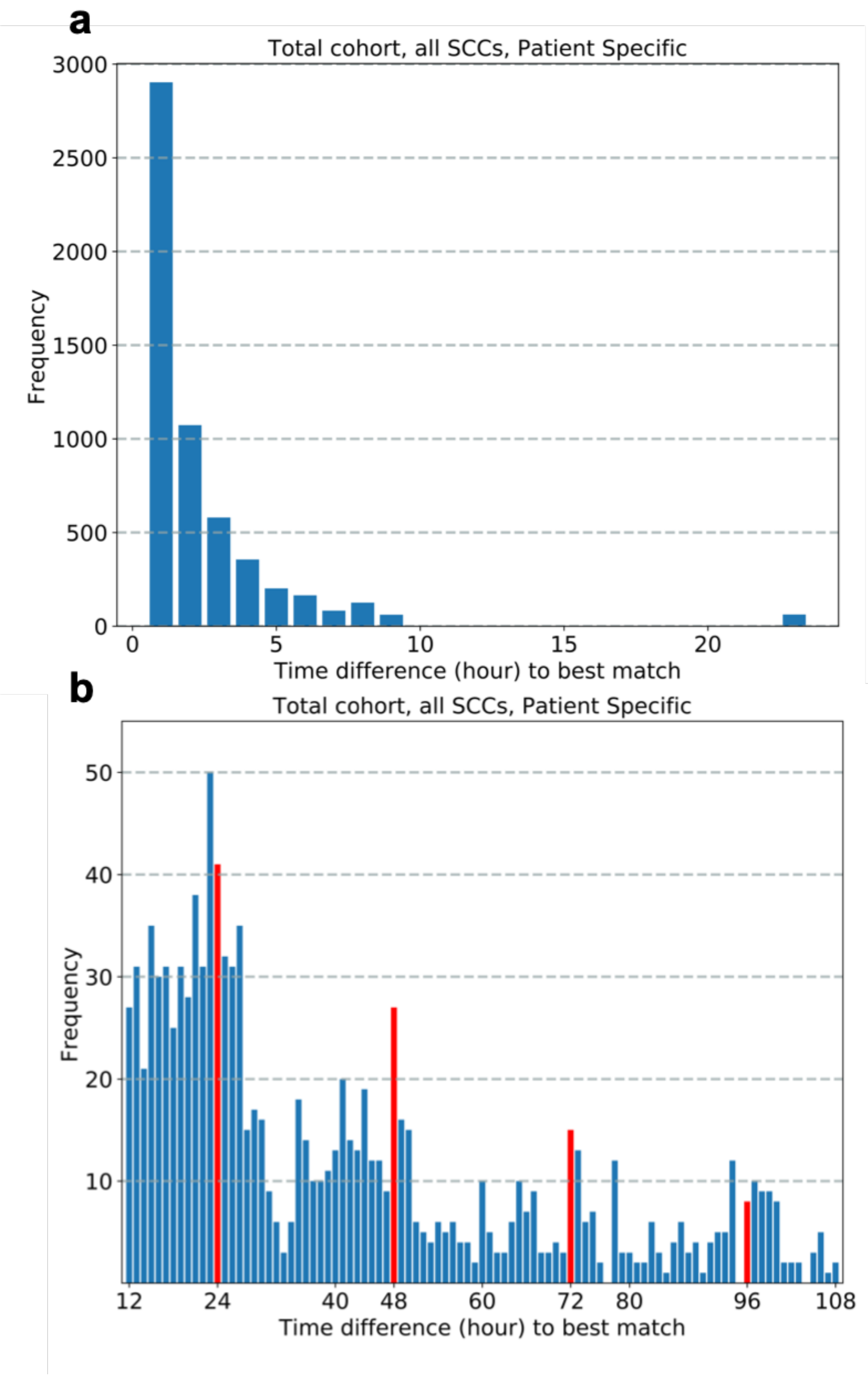

Supplementary Figure 4

(a) Histogram of the ten most frequent time differences (in hours) between a regular hour in the test set and its best match in the reference set for the patient-specific approach considering all SCC from the total cohort. There is no overlap between hours in test set and hours in the reference set. (b) Time difference to best match with minimum time gap of 12h, illustrating that best matches can be temporally distant. The expected higher correlation due to the circadian rhythm of humans is visualized by red bars.

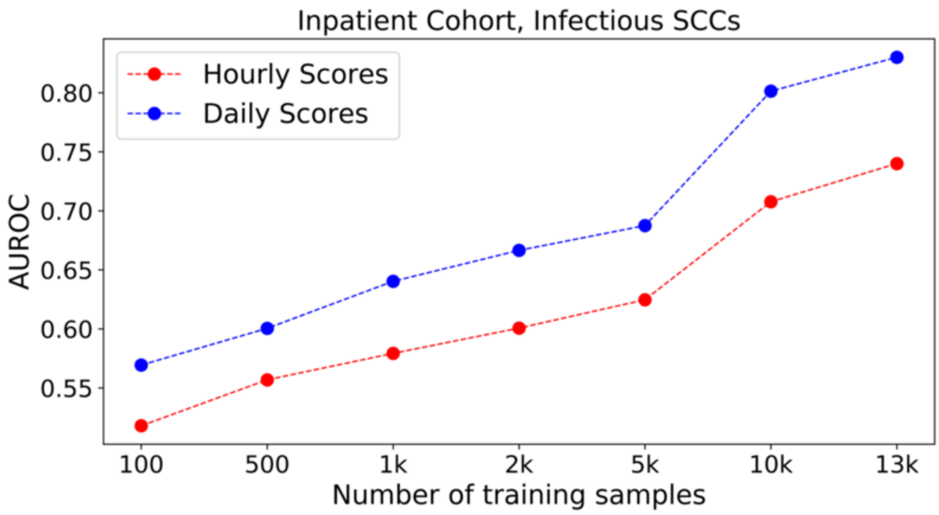

Supplementary Figure 5

SCC-Score performance evaluated using AUROC for different training set sizes. Shown are hourly scores (red dashed line) and daily scores (blue dashed line) for infectious SCCs of the Inpatient Cohort.
